# Optical coherence tomography in myelin-oligodendrocyte-glycoprotein antibody-seropositive patients: a longitudinal study

**DOI:** 10.1186/s12974-019-1521-5

**Published:** 2019-07-25

**Authors:** Frederike C. Oertel, Olivier Outteryck, Benjamin Knier, Hanna Zimmermann, Nadja Borisow, Judith Bellmann-Strobl, Astrid Blaschek, Sven Jarius, Markus Reindl, Klemens Ruprecht, Edgar Meinl, Reinhard Hohlfeld, Friedemann Paul, Alexander U. Brandt, Tania Kümpfel, Joachim Havla

**Affiliations:** 1 Experimental and Clinical Research Center, Max Delbrueck Center for Molecular Medicine and Charité – Universitätsmedizin Berlin, Freie Universität Berlin, Humboldt-Universität zu Berlin and Berlin Institute of Health, Robert-Rössle-Straße 10, 13125 Berlin, Germany; 2 NeuroCure Clinical Research Center, Charité – Universitätsmedizin Berlin, Freie Universität Berlin, Humboldt-Universität zu Berlin and Berlin Institute of Health, Charitéplatz 1, 10117 Berlin, Germany; 3 Department of Neurology and Neuroradiology, Roger Salengro Hospital, University of Lille, INSERM 1171, Avenue du Professeur Emile Laine, 59037 Lille, France; 4 Department of Neurology, Klinikum Rechts der Isar, Technische Universität München, Ismaninger Straße 22, 81675, Munich, Germany; 5 Munich Cluster for Systems Neurology, Feodor-Lynen-Str 17, 81377 Munich, Germany; 6 Department of Pediatric Neurology and Developmental Medicine, Dr. von Hauner’s Children’s Hospital, University of Munich, Lindwurmstraße 4, 80337 Munich, Germany; 7 Molecular Neuroimmunology Group, Department of Neurology, University of Heidelberg, Im Neuenheimer Feld 400, 69120 Heidelberg, Germany; 8 Clinical Department of Neurology, Medical University of Innsbruck, Anichstraße 35, 6020 Innsbruck, Austria; 9 Department of Neurology, Charité – Universitätsmedizin Berlin, Freie Universität Berlin, Humboldt-Universität zu Berlin and Berlin, Institute of Health, Charitéplatz 1, 10117 Berlin, Germany; 10 Institute of Clinical Neuroimmunology, Ludwig-Maximilians University, Marchioninistr. 15, 81377 Munich, Germany; 11 Department of Neurology, University of California Irvine, 30, 101 The City Dr S, Orange, CA 92868 USA; 12 Data Integration for Future Medicine consortium (DIFUTURE), Ludwig-Maximilians University, Marchioninistr. 15, Munich, 81377 Germany

**Keywords:** Optical coherence tomography, Optic neuritis, Myelin-oligodendrocyte-glycoprotein

## Abstract

**Background:**

Serum antibodies against myelin-oligodendrocyte-glycoprotein (MOG-IgG) are detectable in a proportion of patients with acute or relapsing neuroinflammation. It is unclear, if neuro-axonal damage occurs only in an attack-dependent manner or also progressively. Therefore, this study aimed to investigate longitudinally intra-retinal layer changes in eyes without new optic neuritis (ON) in MOG-IgG-seropositive patients.

**Methods:**

We included 38 eyes of 24 patients without ON during follow-up (F/U) [median years (IQR)] 1.9 (1.0–2.2) and 56 eyes of 28 age- and sex-matched healthy controls (HC). The patient group’s eyes included 18 eyes without (Eye^ON-^) and 20 eyes with history of ON (Eye^ON+^). Using spectral domain optical coherence tomography (OCT), we acquired peripapillary retinal nerve fiber layer thickness (pRNFL) and volumes of combined ganglion cell and inner plexiform layer (GCIP), inner nuclear layer (INL), and macular volume (MV). High-contrast visual acuity (VA) was assessed at baseline.

**Results:**

At baseline in Eye^ON-^, pRNFL (94.3 ± 15.9 μm, *p* = 0.36), INL (0.26 ± 0.03 mm^3^, *p* = 0.11), and MV (2.34 ± 0.11 mm^3^, *p* = 0.29) were not reduced compared to HC; GCIP showed thinning (0.57 ± 0.07 mm^3^; *p* = 0.008), and VA was reduced (logMAR 0.05 ± 0.15 vs. − 0.09 ± 0.14, *p* = 0.008) in comparison to HC. Longitudinally, we observed pRNFL thinning in models including all patient eyes (annual reduction − 2.20 ± 4.29 μm vs. − 0.35 ± 1.17 μm, *p* = 0.009) in comparison to HC. Twelve Eye^ON-^ with other than ipsilateral ON attacks ≤ 6 months before baseline showed thicker pRNFL at baseline and more severe pRNFL thinning in comparison to 6 Eye^ON-^ without other clinical relapses.

**Conclusions:**

We observed pRNFL thinning in patients with MOG-IgG during F/U, which was not accompanied by progressive GCIP reduction. This effect could be caused by a small number of Eye^ON-^ with other than ipsilateral ON attacks within 6 months before baseline. One possible interpretation could be a reduction of the swelling, which could mean that MOG-IgG patients show immune-related swelling in the CNS also outside of an attack’s target area.

**Electronic supplementary material:**

The online version of this article (10.1186/s12974-019-1521-5) contains supplementary material, which is available to authorized users.

## Background

Antibodies against conformation-dependent epitopes of myelin-oligodendrocyte-glycoprotein (MOG-IgG) have been described in patients with central nervous system (CNS) inflammation of putative autoimmune etiology [1–4]. MOG is also the dominant antigen for demyelinating antibodies in experimental autoimmune encephalomyelitis (EAE), the predominant animal model of multiple sclerosis (MS), and MOG-IgG can augment demyelination by cell-mediated and humoral immune responses [1]. In neuropathology studies, MOG-IgG are associated with MS-like pathology directed against myelin and oligodendrocytes and biopsies present a MS pattern II [5, 6]. MOG-IgG affinity-purified from the blood of patients with optic neuritis (ON) enhanced inflammation and induced demyelination upon transfer into experimental animals indicating the pathogenic potential of MOG-IgG detected in the blood of these patients [7]. It is discussed whether MOG-IgG define a separate disease entity tentatively called MOG-IgG-associated diseases, MOG-IgG autoimmunity or MOG-IgG seropositive encephalomyelitis rather than being part of several autoimmune disorders, especially neuromyelitis optica spectrum disorders (NMOSD) [1, 3, 8, 9]. However, the bouquet of clinical phenotypes in MOG-IgG-associated diseases at clinical onset is not easy to differentiate and overlaps with aquaporin-4-IgG (AQP4-IgG)-seropositive NMOSD and in rare cases with MS [2, 10–12], although distinct clinical features such as seizures have been described [13–15]. ON is the most common manifestation and can lead to substantial neuro-axonal damage after multiple relapses, as shown in different cohorts [11, 16]. The pattern of retinal degeneration after ON seems to be similar in all MOG-IgG-seropositive cohorts as shown by optical coherence tomography (OCT) studies [11, 16]. OCT proved to be a precise and reproducible method for non-invasive visualization and quantification of retinal layers and plays a crucial role in analyzing retinal changes in various neuroinflammatory disorders [17–20]. In a cross-sectional study, MOG-IgG-related OCT features indicated subclinical pathology in eyes without a history of ON (Eye^ON-^) [16]. However, no longitudinal OCT data is reported in MOG-IgG-associated diseases so far and the pattern of longitudinal retinal damage still remains elusive. Using OCT, we assessed retinal layer thinning as a marker of neuro-axonal damage in a cohort of MOG-IgG-seropositive patients without ON during follow-up (F/U). We aimed to investigate at baseline and longitudinally microstructural changes in MOG-IgG-seropositive patients, extending previous work in AQP4-IgG-seropositive NMOSD [21, 22].

## Methods

### Study populations

Twenty-four patients were seen and followed [F/U (years; median (inter-quartile-range (IQR))) 1.9 (1.0–2.2)] at four university tertiary care centers specialized in neuroimmunological diseases (Institute of Clinical Neuroimmunology, Ludwig Maximilians University (LMU), Munich, Germany, *N* = 11; NeuroCure Clinical Research Center, Charité – Universitätsmedizin Berlin, Germany, *N* = 10; Department of Neurology, University of Lille Hospital, Lille, France, *N* = 1; Department of Neurology, Klinikum Rechts der Isar, Technische Universität München (TUM), Munich, Germany, *N* = 2). Written informed consent was obtained from all patients participating in the study. The local ethics committees approved the study protocol in accordance with the Declaration of Helsinki (1964) in its currently applicable version. All patients were matched by age (*W* = 370, *p* = 0.542) and sex (*χ*^2^ = 0,937, *p* = 0.333) to 56 eyes of 28 healthy controls (HC; F/U) [years; median (IQR)] 1.9 (1.9–2.3) from the NeuroCure Clinical Research Center, Charité – Universitätsmedizin Berlin, Germany. Inclusion criteria were the detection of MOG-IgG, complete longitudinal clinical and OCT imaging data with minimum F/U of  8 months and age between 15 and 75 years at baseline. Only eyes without concomitant potentially confounding diseases (glaucoma, diabetes mellitus, retinal surgery, retinal disease, ametropia > 6 diopters) were included. Eyes with a history of ON ≤ 5 months before baseline were excluded. Clinical data (diagnosis, disease onset, number of ON, date last ON, brain attacks, myelitis, EDSS, relapses, treatment history) were collected for all patients. For detection of MOG-IgG, serum samples from all patients were analyzed at least once by established cell-based assays at the discretion of each center using the laboratory’s cutoffs (MOG IFT, EUROIMMUN, Laboratory Stöcker, Germany; Molecular Neuroimmunology Group, University Heidelberg, Heidelberg, Germany; Reindl Lab, Medical University of Innsbruck, Innsbruck, Austria; Meinl Lab, LMU, Munich, Hemmer Lab, TUM, Munich) [3, 7, 23].

### Optical coherence tomography

All centers used SPECTRALIS spectral-domain OCT (Heidelberg Engineering, Heidelberg, Germany) with automatic real-time (ART) function for image averaging. We acquired peripapillary retinal nerve fiber layer thickness (pRNFL) and volumes of combined ganglion cell and inner plexiform layer (GCIP), inner nuclear layer (INL) and macular volume (MV) by OCT. GCIP, INL and MV were calculated as a 3 mm diameter cylinder around the fovea from a macular volume scan (25° × 30°, 61 vertical B-scans, 12 ≤ ART ≤ 18; 20° × 20°, 25 vertical B-scans, 27 ≤ ART ≤ 49). The peripapillary RNFL (pRNFL) was measured with activated eye tracker using ring scans around the optic nerve (12°, 1536 A-scans, 57 ≤ ART ≤ 100) or the most inner ring of a star-and-ring scan around the optic nerve (12°, 768 A-scans, 27 ≤ ART ≤ 33). For two patients (8.3%), the ring scan protocol changed during the acquisition period (ring scan to inner ring of a star-and-ring scan)*.* Segmentation of all layers was performed semi-automatically using software provided by the OCT manufacturer (Eye Explorer 1.9.10.0 with viewing module 6.3.4.0, Heidelberg Engineering, Heidelberg, Germany). Experienced raters (BK for TU Munich data, JH for all other data) carefully checked all scans for sufficient quality and segmentation errors and corrected if necessary. OCT data in this study is reported and analyzed according to the APOSTEL and OSCAR-IB recommendations [24, 25]. Macular microcysts were defined as the presence of cystic lesions on at least one scan detected by experienced raters (BK for TU Munich scans, JH for all other scans). Additionally, we collected habitually corrected monocular high-contrast visual acuity (VA) using ETDRS (Early Treatment Diabetic Retinopathy Study) charts at baseline in 20 ft distance for a subset of patients (*N* = 15).

### Statistical methods

Group differences between MOG-IgG patients and HC were tested by chi-squared test for sex and Wilcoxon rank-sum test for age. Main outcomes were change of GCIP, pRNFL, INL and MV and VA over F/U. Cross-sectional differences of OCT values and VA between all groups were analyzed pairwise by generalized estimating equation (GEE) models to account for inter-eye within-patient correlations of monocular measurements. Longitudinal analyses of OCT and VA were performed with linear mixed effects models using time from baseline and group as fixed effects and patient-ID and eye-ID as random effects; results are reported for effect “Time from Baseline * Group”, which reflects the group-specific change over time. Annual loss was estimated for each individual as change to baseline at last visit divided by F/U time in years. All tests and graphical representations were performed with R version 3.3.1 [http://www.R-project.org]. Statistical significance was established at *p* < 0.05, and all results were interpreted in the context of an exploratory analysis and therefore not adjusted for multiple comparison.

## Results

### Cohort description and follow-up

We included 38 eyes of 24 patients without ON during F/U. 70% of the patients from Berlin [7/10] and 64% of the patients from LMU Munich [7/11] have been included in previous cross-sectional studies [7, 10, 16]. MOG-IgG-seropositive patients had the following diagnosis: recurrent ON (*N* = 7), MOG-IgG-seropositive NMOSD (*N* = 12) meeting the 2015 IPND (International Panel for Neuromyelitis Optica Diagnosis) criteria for seronegative NMOSD [26], MOG-IgG-seropositive MS (*N* = 3) and MOG-IgG-seropositive meningoencephalomyelitis (*N* = 2). All patients had ≥ 1 F/U visit(s) [median (range) 2 visits (2–7)]. The MOG-IgG-seropositive cohort included 18 eyes without (Eye^ON-^) and 20 eyes with a history of ON (Eye^ON+^) (number of ONs [median (range)] 0 (0 – 8); time since ON in years [median (range)] 2.2 (0.4 – 14.9)). From the 18 Eye^ON-^, we identified 12 eyes with other than ipsilateral ON attacks within 6 months before baseline (five eyes of three patients with a myelitis, four eyes of two patients with myelitis and brainstem attacks, one eye of one patient with myelitis and contralateral ON and 2 eyes of 2 patients with contralateral ON; age 40 ± 9, male/female 5/3, EDSS 2.5, median follow-up 14 ± 5.9 months)) and six eyes without other attacks (age 39.0 ± 21.0, male/female 1/4, EDSS 3.5, median follow-up 26 ± 4.5 months). Retrospectively, one patient (2 eyes) could not be included in the study analysis because he had ONs on both sides during F/U and another patient (2 eyes) could not be included because he had insufficient follow-up less than 8 months. Data of further 8 eyes had to be excluded (five eyes with ON during F/U, one eye with ON less than 5 months before study inclusion, one eye with missing data, one eye with OCT-confounding disease). Clinical characteristics of all included patients are shown in Table 1.

**Table 1:** Clinical characteristics of patients. Age (*W* = 370, *p* = 0.542) and sex (*χ*^2^ = 0.937, *p* = 0.333) did not differ between MOG-IgG-seropositive patients and HCs

	HC	MOG-IgG-seropositive patients
Subjects [*N*]	28	24
Number of eyes [*N*]	56	38
F/U [median years (min, max)]	1.9 (0.8–3.3)	1.9 (0.6–2.8)
Age [mean (SD)]; [range at baseline]	43.12 (9.76); [11–68]	40.66 (13.53); [15–68]
Sex [male (%)]	6 (21.4)	9 (37.5)
Clinical phenotypes (MOG-IgG-associated diseases)	–	ON (*N* = 7), NMOSD (*N* = 12), MS (*N* = 3), meningoencephalomyelitis (*N* = 2)
EDSS at baseline [median (IQR)]	–	2.5 (2.0; 3.0)
Disease duration at baseline in years [median (IQR)]	–	3.0(1.1; 8.8)
Eyes with a history of ON [Eye^ON+^, *N* (%)]	–	20 (52.6%)
Patients with a history of ON [*N* (%)]	–	15 (62.5%)
Number of ON in Eye^ON+^ [median (range)]	–	2 (1–8)
Eyes without a history of ON [Eye^ON-^, *N* (%)]	–	18 (47.4%)
Time since ON [years; median (range)]	–	2.2 (0.4–14.9)
Eyes with contralateral ON during F/U [*N* (%)]	–	5 (13.2)
Treatment at baseline [*N*]	–	AZA [4], MTX [1], NAT [1], RIX [8], IVIG [1], PRED [2], NONE [7]

*Abbreviations*: *HC* healthy controls, *N* number, *SD* standard deviation, *F/U* follow-up, *AZA* azathioprine, *MTX* methotrexate, *NAT* natalizumab, *RIX* rituximab, *IVIG* intravenous immunoglobulins, *PRED* prednisone, *TOC* tocilizumab, *MMF* mycophenolate mofetil, *NONE* no treatment

### Group differences at baseline

**Fig. 1:**
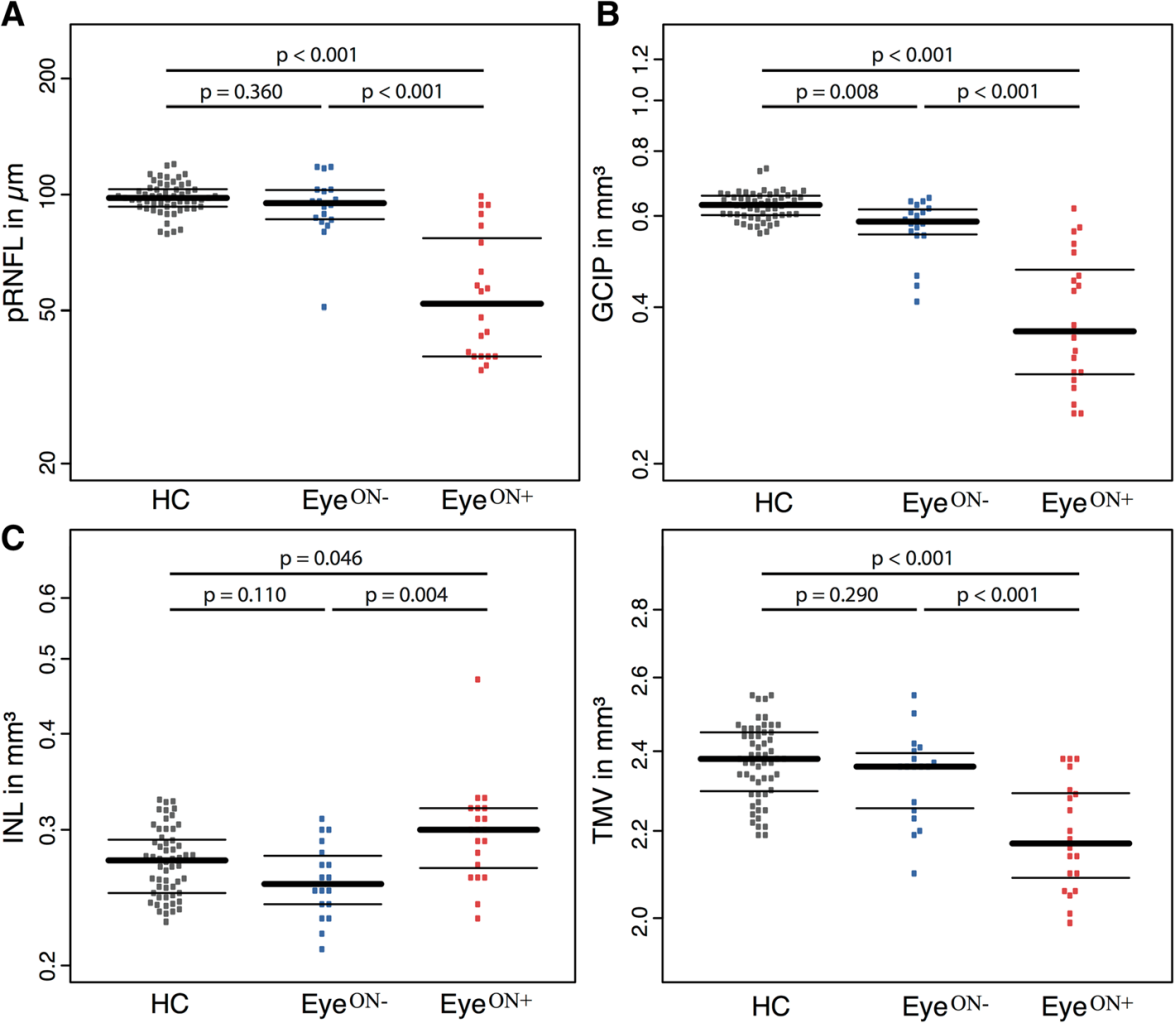
Baseline data: bee swarm plots of cross-sectional OCT data for HC (gray, left), MOG-IgG-seropositive Eye^ON-^ (blue, middle) and MOG-IgG-seropositive Eye ^ON+^ (red, right) (median ± IQR, single eyes as dots) for **a** pRNFL, **b** GCIP, **c** INL, and **d** MV. Abbreviations: Eye ^ON-^: MOG-IgG-seropositive eyes without a history of ON; Eye ^ON+^: MOG-IgG-seropositive eyes with a history of ON; GCIP: combined ganglion cell and inner plexiform layer; HC: Healthy control; INL: inner nuclear layer; IQR: inter-quartile range; OCT: Optical coherence tomography; p: *p* value; pRNFL: peripapillary retinal nerve fiber layer; MV: macular volume

First, we analyzed group differences at baseline between MOG-IgG-seropositive patient eyes with a history of ON (Eye^ON+^), patient eyes without previous ON (Eye^ON-^) and eyes from HC. At baseline, in Eye^ON-^, pRNFL, INL and MV were not significantly different, but GCIP was significantly thinner in comparison to HC (*p* = 0.008) (Table 2, Fig. 1). VA was lower in Eye^ON-^ in comparison to HC (*p* = 0.013).

**Table 2:** Baseline OCT results of MOG-IgG-seropositive patients and HCs

Baseline results	HC	MOG-IgG-seropositive patients Eye^ON-^	MOG-IgG-seropositive patients Eye^ON+^	HC vs Eye^ON-^			HC vs Eye^ON+^			Eye^ON-^ vs Eye^ON+^		
	*N* (eyes) = 56	*N* (eyes) = 18	*N* (eyes) = 20	[*B*]	[SE]	[*p*]	[*B*]	[SE]	[*p*]	[*B*]	[SE]	[*p*]
GCIP [mean (SD)]	0.63 (0.04)	0.57 (0.07)	0.39 (0.12)	− 0.057	0.022	*0.008*	− 0.235	0.033	*< 0.0001*	− 0.178	0.037	*< 0.0001*
pRNFL [mean (SD)]	98.50 (9.17)	94.33 (15.92)	58.25 (22.56)	− 4.167	4.577	0.360	− 40.25	5.864	*< 0.0001*	− 36.08	6.311	*< 0.0001*
INL [mean (SD)]	0.27 (0.03)	0.26 (0.03)	0.30 (0.05)	− 0.014	0.009	0.110	0.026	0.013	*0.046*	0.041	0.014	*0.004*
MV [mean (SD)]	2.37 (0.10)	2.34 (0.11)	2.19 (0.13)	− 0.036	0.034	0.290	− 0.183	0.039	*< 0.0001*	− 0.147	0.042	*0.0005*
HCVA in logMAR [mean (SD)]	−0.09 (0.14)	0.05 (0.15)	0.55 (0.81)	0.146	0.059	*0.013*	0.394	0.134	*0.0032*	0.248	0.131	0.058

*Abbreviations*: *B* estimate, *GCIP* combined ganglion cell and inner plexiform layer, *HC* healthy control, *INL* inner nuclear layer, *Eye*^*ON-*^ MOG-IgG-seropositive patients without a history of ON, *Eye*^*ON+*^ MOG-IgG-seropositive patients with a history of ON, *OCT* optical coherence tomography, *ON* optic neuritis, *p p* value, *pRNFL* peripapillary retinal nerve fiber layer, *SD* standard deviation, *SE* standard error, *MV* macular volume, *vs* versus, *N* number of eyes

In Eye^ON+^ at baseline pRNFL, GCIP and MV were significantly lower in comparison to HC (pRNFL *p*  < 0.0001, GCIP *p*  < 0.0001, MV *p*  < 0.0001). In contrast, INL was significantly thicker in Eye^ON+^ (INL 0.30 ± 0.05 mm^3^ vs. 0.27 ± 0.03 mm^3^ (*p* = 0.046)). VA was also lower in Eye^ON+^ (0.55 ± 0.81) in comparison to HC [− 0.09 (0.14), *p* = 0.01) and Eye^ON-^ [0.05 (0.15), *p* = 0.058).

One Eye^ON-^ showed a massive thinness of the pRNFL at baseline despite a missing history of ON. We found macular microcysts within the INL in 6/20 (30%) Eye^ON+^.

### OCT changes during F/U

**Fig. 2:**
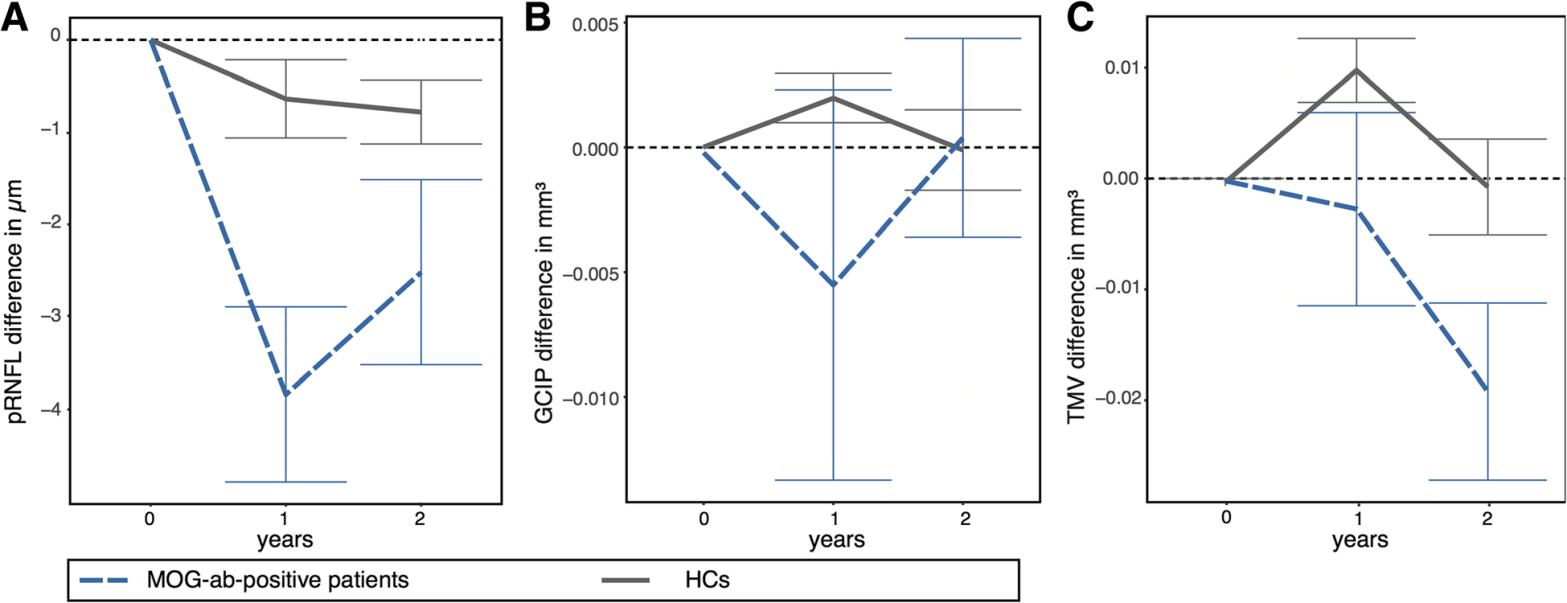
Bar graphs of longitudinal OCT data. Plotted change (mean ± standard error) for rounded time since baseline in years for **a** pRNFL and **b** GCIP, **c** MV for eyes of MOG-IgG-seropositive patients (blue, dashed), and HC (gray, continuous), displayed until median F/U time (2 years). Abbreviations: F/U: follow-up; GCIP: combined ganglion cell and inner plexiform layer; HC: healthy control; INL: inner nuclear layer; Eye ^ON-^: MOG-IgG-seropositive eyes without a history of ON; ON: optic neuritis; OCT: optical coherence tomography; pRNFL: peripapillary retinal nerve fiber layer; SE: standard error, MV: macular volume. F/U investigations were rounded up or down to the year 0, 1, or 2 and follow-up visits with a time since baseline < 6 months were excluded from the graphical display. *N rounded for timepoints:* T0: *N* (MOG) = 38 eyes, T1: *N* (MOG) = 27 eyes, T2: *N* (MOG) = 26 eyes, T0: *N* (HC) = 56 eyes, T1: *N* (HC) = 41 eyes, T2: *N* (HC) = 40 eyes

**Fig. 3:**
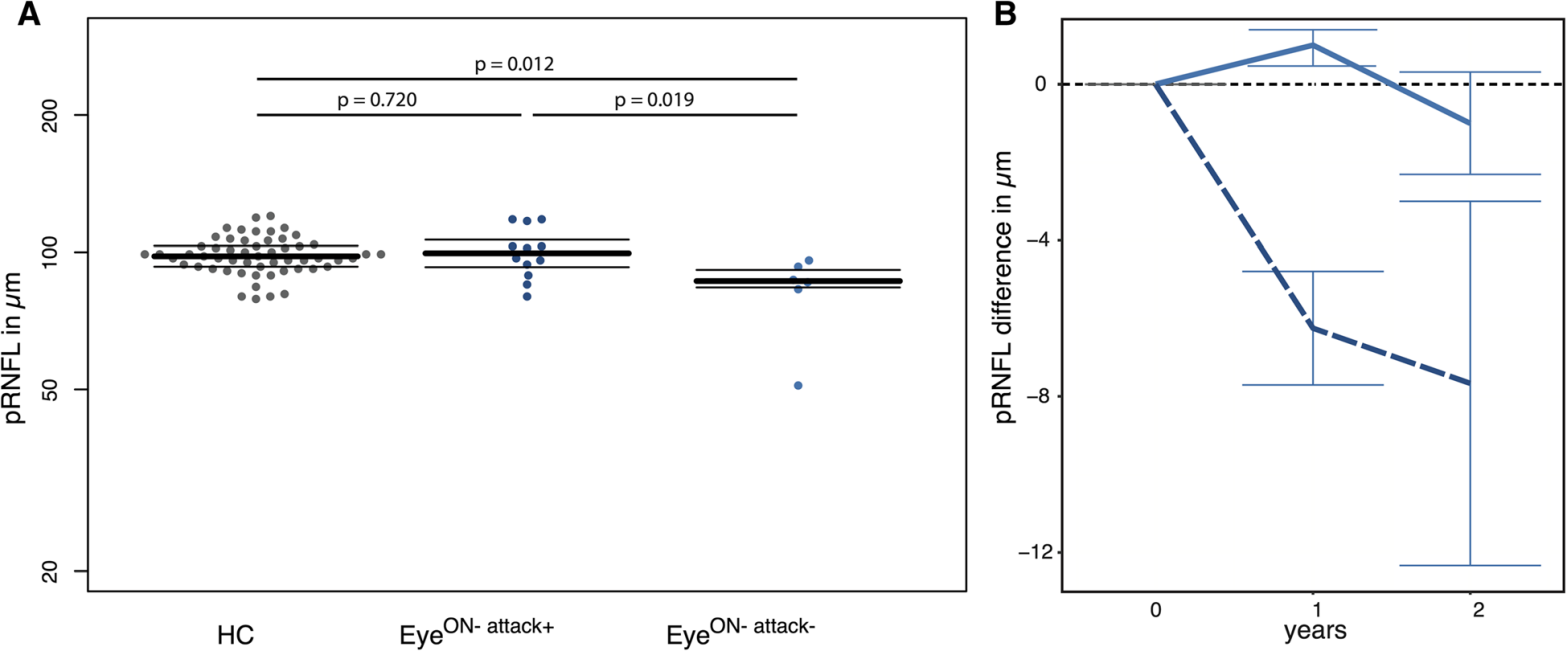
**a** Bee swarm plots of cross-sectional OCT data for HC (gray, left), Eye^ON-^ with non-ipsilateral ON attacks ≤ 6 months before baseline (blue, middle), and Eye^ON-^ with no attacks ≤ 6 months before baseline (blue, right) (median ± IQR, single eyes as dots) for pRNFL. **b** Bar graphs of longitudinal OCT data. Plotted change (mean ± standard error) for rounded time since baseline for pRNFL of Eye^ON-^ with other attacks (blue, dashed) and Eye^ON-^ without other attacks (blue, continuous), displayed until median F/U time. F/U investigations were rounded up or down to the year 0, 1, or 2 and follow-up visits with a time since baseline < 6 months were excluded from the graphical display. *Eye*^*ON-attack-*^ T0/1/2: *N* = 6 eyes, *Eye*^*ON-attack-*^ T0: *N* = 12 eyes, T1: *N* = 11 eyes, T2: *N* = 3 eyes. Abbreviations: F/U: follow-up; HC: healthy control; Eye ^ON-^: MOG-IgG-seropositive eyes without a history of ON; ON: optic neuritis; OCT: optical coherence tomography; pRNFL: peripapillary retinal nerve fiber layer; N: number of eyes that contributed to the analysis

Longitudinally, we observed pRNFL thinning, which was not accompanied by progressive GCIP reduction, in eyes without ON during F/U (annual loss: − 2.20 ± 4.29 μm vs. HC -0.35 ± 1.17 μm, *p* = 0.009) (Fig. 2; individual changes in Additional file 1). There were no longitudinal group differences between Eye^ON+^ and Eye^ON-^ for GCIP, pRNFL, INL and MV as well as between MOG-IgG-seropositive NMOSD and other MOG-IgG-seropositive patients (Table 3). In a previous study investigating spinal cord changes in MOG-IgG patients, we suspected edematous changes in patients close to a clinical attack [27]. We therefore investigated patients with a non-ipsilateral ON attack within 6 months of the baseline visit in a subgroup analysis. At baseline, the pRNFL in 12 Eye^ON-^ with a non-ipsilateral ON attack within the 6 months before baseline was thicker in comparison to 6 Eye^ON-^ without a non-ipsilateral ON attack within the 6 months before baseline (pRNFL 100.2 ± 12.7 μm vs. 82.7 ± 16.2 μm (*p* = 0.019)) (Fig. 3A). Reduction of pRNFL thickness was seen mainly in 3 eyes of the subgroup analysis. Two of the 3 eyes had no clinical evidence of unilateral ON attacks of the contralateral eye within the 6 months prior to inclusion in the study. One of the 3 eyes had a relapse complex with myelitis, brain attack and contralateral ON within 6 months prior to baseline. An ON-affection of these 12 Eye^ON-^ with a non-ipsilateral ON attack was further ruled out by a stable high-contrast visual acuity (HCVA) without a change during F/U (HCVA as decimal, median (range): at baseline 1.0 (0.6–1.1); at last visit 1.0 (0.6–1.6)). A longitudinal graphical display of Eye^ON-^ showed the pRNFL thinning to be predominantly present in Eye^ON-^ with an attack before baseline (Fig. 3B). However, due to the small sample size, no statistical analysis could be performed.

**Table 3:** Longitudinal OCT results of MOG-IgG-seropositive patients and HCs

Longitudinal OCT data	HC	MOG-IgG-seropositive patients Eye^ON-^	MOG-IgG-seropositive patients Eye^ON+^	HC vs MOG-IgG-seropositive patients (Eye^ON-^ and Eye^ON+^)				Eye^ON-^ vs Eye^ON+^			
	Absolute change to baseline										
	*N* (eyes) = 56	*N* (eyes) = 18	*N* (eyes) = 20	[*B*]	[95%CI]	[SE]	[*p*]	[*B*]	[95%CI]	[SE]	[*p*]
GCIP [mean (SD)]	0.00 (0.01)	0.00 (0.01)	0.00 (0.05)	− 0.000	− 0.004; 0.005	0.002	0.884	− 0.006	− 0.016; 0.003	0.005	0.214
pRNFL [mean (SD)]	− 0.61 (2.00)	− 4.5 (5.89)	− 1.60 (4.48)	− 1.645	− 2.819; − 0.471	0.599	*0.009*	0.168	− 1.380; 1.717	0.790	0.832
INL [mean (SD)]	0.00 (0.01)	0.01 (0.02)	0.00 (0.03)	0.002	−0.002; 0.005	0.002	0.312	− 0.003	− 0.011; 0.004	0.004	0.381
MV [mean (SD)]	0.00 (0.02)	− 0.01 (0.04)	− 0.01 (0.05)	− 0.008	− 0.016; 0.0013	0.004	0.103	0.002	− 0.013; 0.018	0.008	0.769

*Abbreviations*: *95%CI* 95% confidence interval, *B* Estimate (beta-coefficient), *GCIP* combined ganglion cell and inner plexiform layer, *HC* healthy control, *INL* inner nuclear layer, *Eye*^*ON-*^ MOG-IgG-seropositive patients without a history of ON, *Eye*^*ON+*^ MOG-IgG-seropositive patients with a history of ON, *OCT* optical coherence tomography, *p p* value, *pRNFL* peripapillary retinal nerve fiber layer, *SD* standard deviation, *SE* standard error, *MV* macular volume, *vs* versus, *N* number of eyes

## Discussion

In this study, we investigated longitudinally, MOG-IgG-seropositive patients for potential progressive or covert damage in the retina in the absence of new clinical ON. We could not detect progressive GCIP thinning during F/U in MOG-IgG-seropositive patients, which is in contrast to progressive GCIP reduction in AQP4-IgG-seropositive NMOSD and MS [22, 27]. Instead, we observed a longitudinal pRNFL reduction, which in a consequent subgroup analysis appeared to primarily occur in patients with non-ipsilateral ON attacks within 6 months before baseline. A hypothetical explanation of this finding could be a remission of pRNFL edema.

Cross-sectional retinal imaging studies have shown conflicting results as to whether MOG-IgG-associated diseases have a more favorable outcome compared to patients with ON in other disease contexts [28–34]. The presumed higher relapse rates in MOG-IgG-seropositive patients could be associated with a severe retinal neuroaxonal loss and an unfavorable visual outcome [11]. Although OCT data regarding MOG-IgG-associated retinal damage are inconsistent [11, 16, 30, 35], neuroaxonal retinal damage may occur as a consequence of clinical episode(s) of ON or of subclinical involvement [11, 16]. ON was associated with macular microcysts, a biomarker suggestive of severe optic neuropathy [16, 36, 37]. A previous study investigating a smaller cohort of MOG-IgG-positive patients showed a significant reduction of the pRNFL and the ganglion cell layer in Eye^ON-^ compared to HC cross-sectionally [16]. By contrast, in our current study, we could only confirm a significant GCIP reduction in Eye^ON-^ at baseline but no significant reduction of the pRNFL as a hint towards subclinical retinal pathology. However, pRNFL edema as a marker of immune-related swelling in the CNS after relapses and also outside of relapses could have contributed to this finding. The GCIP reduction at baseline could be discussed as progressive neurodegenerative retinal involvement, subclinical optic nerve pathology, chiasmal crossover of ON in contralateral eyes, or as an expression of subclinical ON in the previous patient’s history. However, according to Ramanathan et al., only 5% of ONs in MOG-IgG-seropositive patients shows chiasmal involvement [38].

Longitudinally, we observed pRNFL but not GCIP thinning. We hypothesize that this can be explained not only by subclinical retinal or optic nerve involvement or drug-induced retinal damage related to immunosuppressive treatment, but also by a remission of non-ipsilateral ON attacks that has occurred in Eye^ON-^ within 6 months before baseline since patients without clinical attacks ≤ 6 months before baseline did not present significant pRNFL or GCIP loss during F/U. This is clearly in contrast to our recently published data about longitudinal GCIP thinning in AQP4-IgG-seropositive NMOSD [22] or earlier studies reporting GCIP loss in MS [27] and might be an important hint towards the differentiation of MOG-IgG-associated diseases from AQP4-IgG-seropositive NMOSD. AQP4-IgG-seropositive NMOSD is an astrocytopathy, and a primary retinopathy caused by antibody-mediated damage is supported by animal studies and recently also clinical studies [22, 39]. In contrast, the retina does not harbor myelin-producing oligodendrocytes and an expression of MOG has not been shown, making a primary retinopathy unlikely.

Further, data showing clear differences between AQP4-IgG-seropositive NMOSD and MOG-IgG-associated diseases were presented recently by Chien et al. [40]. Spinal cord imaging data showed differences in spinal cord affection patterns and disability accumulation. A higher prevalence of myelitis with clinical attacks and chronic spinal cord lesions was detected for AQP4-IgG-seropositive NMOSD patients in comparison to MOG-IgG-associated diseases [40]. Interestingly, MOG-IgG-seropositive patients showed a swelling of the upper cervical cord area during other non-myelitis attacks, also pointing towards a systemic inflammatory affection in MOG-IgG-associated diseases as potentially shown here in the pRNFL during different attacks [40]. Our data is in line with the conclusion that AQP4-IgG-seropositive NMOSD and MOG-IgG-associated diseases are distinct immunological disorders, but share common clinical patterns [22, 40–42].

Limitations of our study are the heterogeneity of MOG-IgG-seropositive patients with different clinical phenotypes in our cohort, the heterogeneity of immunosuppressive treatments of our patients, and due to the rarity of MOG-IgG-seropositive patients in Europe, the small sample size, which leads to outliers possibly having a larger effect on the results, short and variable F/U, and the evaluation of MOG-IgG by different labs using different assays. Additionally, our study lacks magnetic resonance imaging data on optic nerve lesion lengths and lesion volumes of the afferent visual system as well as whole-brain lesion volume to further evaluate subclinical retinal atrophy in MOG-IgG-associated diseases.

## Conclusions

We report in this small explorative study of MOG-IgG-associated diseases no evidence of GCIP thinning during F/U. Additionally, we found pRNFL reduction without GCIP loss during F/U predominantly in Eye^ON-^ with other than ipsilateral ON attacks ≤ 6 months before baseline. We will investigate in a planned longitudinal study involving more centers, whether this reduction is actually due to a remission of edema or reflects retinal neurodegenerative processes or drug-induced retinal damage related to aggressive immunosuppressive treatment.

## Additional file

Additional file 1: **Figure S1.** Spaghetti plots of longitudinal OCT data. Plotted absolute values of all subjects for time since baseline in years for (A) pRNFL and (B) GCIP, (C) MV for eyes of MOG-IgG-seropositive patients (turquoise) and HC (red), as well as (D) plotted absolute values of all Eyes^ON-^ with (green) and without (red) an attack in the 6 months before baseline. Abbreviations: GCIP: Combined ganglion cell and inner plexiform layer, HC: Healthy control, Eye ^ON-^: MOG-IgG-seropositive eyes without a history of ON, ON: Optic neuritis, OCT: Optical coherence tomography, pRNFL: Peripapillary retinal nerve fiber layer, MV: Macular volume. (TIFF 47160 kb)

## Data Availability

The datasets used and/or analyzed during the current study are available from the corresponding author on reasonable request.

## Abbreviations

AQP4-IgG: Aquaporin-4 antibodies
ART: Automatic real time
B: Estimate
CNS: Central nervous system
EAE: Experimental autoimmune encephalomyelitis
EDSS: Expanded disability status scale
Eye^ON-^: Eyes without a history of optic neuritis
Eye^ON+^: Eyes with a history of optic neuritis
F/U: Follow-up
GCIP: Combined ganglion cell and inner plexiform layer
GEE: Generalized estimated eq.
HC: Healthy control
INL: Inner nuclear layer
IQR: Inter-quartile range
LMU: Ludwig-Maximilians University Munich
MOG-IgG: Myelin-oligodendrocyte glycoprotein antibodies
MS: Multiple sclerosis
N: Number
NCRC: NeuroCure Clinical Research Center Berlin
NMOSD: Neuromyelitis optica spectrum disorders
OCT: Optical coherence tomography
ON: Optic neuritis
*p*: *p* value
pRNFL: Peripapillary retinal nerve fiber layer
SD: Standard deviation
SE: Standard error
MV: Macular volume
TUM: Technical University Munich
VA: Visual acuity
