# Supplementary figures and images for: Optical coherence tomography in myelin-oligodendrocyte-glycoprotein antibody-seropositive patients: a longitudinal study
Source: J Neuroinflammation. 2019 Jul 25;16:154. doi: 10.1186/s12974-019-1521-5 (PMC6657100; doi:10.1186/s12974-019-1521-5)

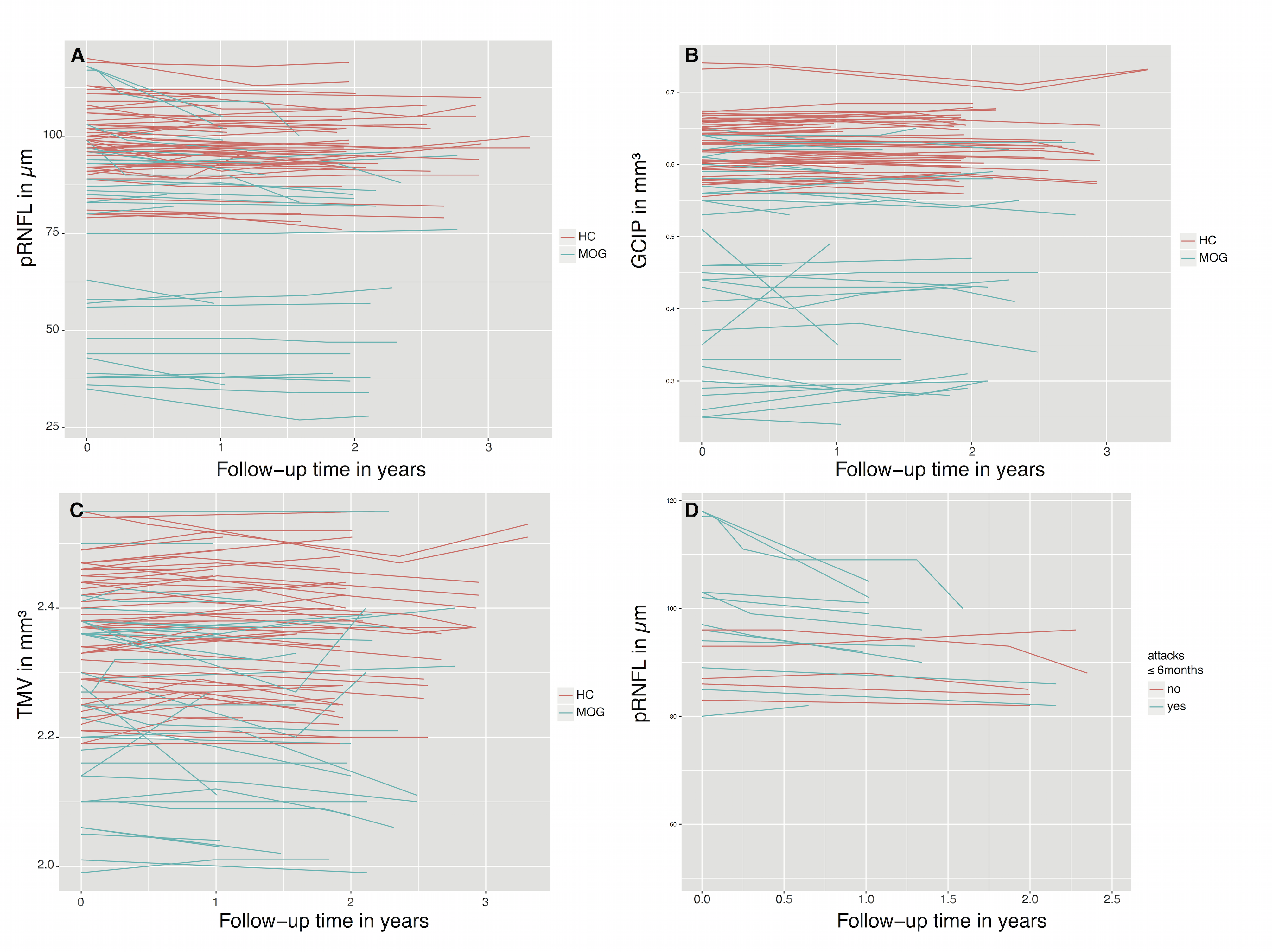

Supplement: Supplementary file 1 — Figure S1. Spaghetti plots of longitudinal OCT data. Plotted absolute values of all subjects for time since baseline in years for (A) pRNFL and (B) GCIP, (C) MV for eyes of MOG-IgG-seropositive patients (turquoise) and HC (red), as well as (D) plotted absolute values of all EyesON- with (green) and without (red) an attack in the 6 months before baseline. Abbreviations: GCIP: Combined ganglion cell and inner plexiform layer, HC: Healthy control, Eye ON-: MOG-IgG-seropositive eyes without a history of ON, ON: Optic neuritis, OCT: Optical coherence tomography, pRNFL: Peripapillary retinal nerve fiber layer, MV: Macular volume. (TIFF 47160 kb) [file 12974_2019_1521_MOESM1_ESM.tiff]
